# Utilizing the *R/V Marcus G. Langseth’s* streamer to measure the acoustic radiation of its seismic source in the shallow waters of New Jersey’s continental shelf

**DOI:** 10.1371/journal.pone.0183096

**Published:** 2017-08-11

**Authors:** Timothy J. Crone, Maya Tolstoy, James C. Gibson, Gregory Mountain

**Affiliations:** 1 Lamont–Doherty Earth Observatory, Columbia University, Palisades, New York, United States of America; 2 Department of Earth and Environmental Sciences, Lamont–Doherty Earth Observatory, Columbia University, Palisades, New York, United States of America; 3 Department of Earth and Planetary Sciences, Rutgers University, Piscataway, New Jersey, United States of America; University of Pavia, ITALY

## Abstract

Shallow water marine seismic surveys are necessary to understand a range of Earth processes in coastal environments, including those that represent major hazards to society such as earthquakes, tsunamis, and sea-level rise. Predicting the acoustic radiation of seismic sources in shallow water, which is required for compliance with regulations designed to limit impacts on protected marine species, is a significant challenge in this environment because of variable reflectivity due to local geology, and the susceptibility of relatively small bathymetric features to focus or shadow acoustic energy. We use data from the *R/V Marcus G. Langseth’s* towed hydrophone streamer to estimate the acoustic radiation of the ship’s seismic source during a large survey of the shallow shelf off the coast of New Jersey. We use the results to estimate the distances from the source to acoustic levels of regulatory significance, and use bathymetric data from the ship’s multibeam system to explore the relationships between seafloor depth and slope and the measured acoustic radiation patterns. We demonstrate that existing models significantly overestimate mitigation radii, but that the variability of received levels in shallow water suggest that in situ real-time measurements would help improve these estimates, and that post-cruise revisions of received levels are valuable in accurately determining the potential acoustic impact of a seismic survey.

## Introduction

Shallow water seismic surveys are critical for understanding a range of geological and oceanographic processes, many of which have the potential to substantially impact human society on both short and long timescales [1]. Some of the processes in this category include large subduction zone earthquakes, tsunamis, and sea-level rise. These events have the potential to displace human populations as well as cause largescale economic disruptions and losses of life. Understanding these processes using active seismic surveys in shallow-water environments can help mitigate the danger these processes pose to society.

At the same time, seismic surveys can potentially influence the behavior of marine mammals [2–4], and raise concerns regarding marine mammal hearing [5] and impacts to fisheries [6]. For these reasons, efforts are taken to minimize the effects of seismic surveys on animal populations. A critical piece of information for this minimization is the estimation of the acoustic radiation of the seismic source towed by the survey vessel. Often this occurs through direct arrival modeling and the extrapolation of data from previous calibration experiments [7–9]. For surveys in deep water, this process is relatively straightforward. However, in shallow-water environments the interaction of acoustic waves with the seafloor and the subbottom makes this process more challenging. In particular, bedform morphology and near-surface stratification can strongly influence received levels from a reflected acoustic source [10]. Furthermore, relatively small changes in slope from minimal bathymetric features can shadow or amplify received levels in spatially variable ways that are difficult to predict, particularly without the availability of high-resolution bathymetric data [11]. A potential solution to this problem is the direct, real-time measurement of the acoustic radiation from a seismic source using the streamer, which is a long towed array of hydrophones deployed as a component of the survey gear.

In this paper we expand on previous efforts to demonstrate the capacity of the multichannel streamer to provide information regarding the acoustic radiation of the seismic source during a shallow-water seismic survey [11]. We use nearly all of the seismic data from a 540 km^2^ survey of the shallow continental shelf off the coast of New Jersey conducted using the *R/V Marcus G. Langseth*, which is the premier seismic vessel of the University-National Oceanographic Laboratory System (UNOLS) fleet. We analyze these data to estimate the received acoustic levels along the streamer and to calculate the distances to acoustic levels used by the National Marine Fisheries Service (NMFS) to regulate the use of seismic equipment in the ocean [12]. We use the bathymetry data collected during the survey using the ship’s multibeam sonar system to explore relationships between the seafloor depth and slope and the measured acoustic radiation patterns.

## The New Jersey multichannel seismic survey

In June of 2015 a multichannel seismic survey was conducted on the central continental shelf off the coast of New Jersey [13, 14] using the *R/V Marcus G. Langseth*. The goal of the survey was to understand the history of local sea-level change and the effects of these changes on the shoreline as recorded in the arrangement of shallow-water sediments, as well as to provide regional context for coring and logging efforts that were undertaken as part of the 2009 IODP Expedition 313 [15, 16].

The survey covered an area approximately 54×10 km^2^, in water depths ranging from about 27–64 m. The local seabed consists primarily of sand-prone sediment, and has a morphology dominated by sand ridges and fossil dunes with amplitudes generally ranging from 1–10 m and widths ranging from 1–5 km [17]. The southeastern part of the survey area includes a ~20-m mid-shelf scarp which in the past was interpreted as a fossil shoreline [17–22]. However, a more recent investigation has shown that this area contains depositional features suggesting that this scarp is more likely the seaward edge of a Hudson River delta lobe [23].

**Fig 1:**
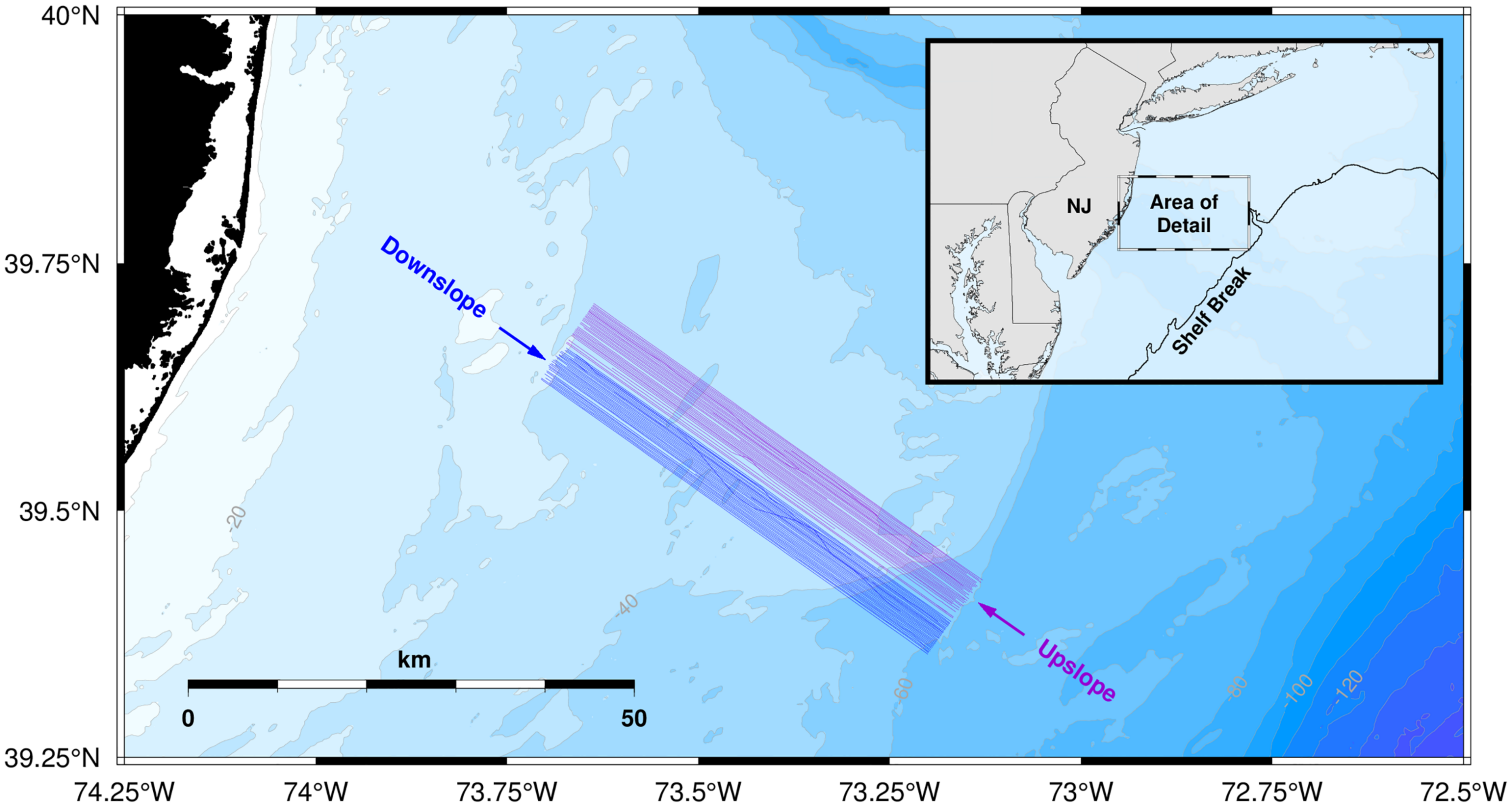
Map of the survey area and survey lines. The inset map shows the location of the survey area relative to the coastline of New Jersey (NJ) and the shelf break. The survey lines in the northern part of the survey area were collected with the ship transiting in the upslope direction. The set of lines in the southern part of the survey area were collected in the downslope direction.

The survey included 79 seismic lines in total, about half of which were collected with the ship transiting in the upslope direction, and the other half in the downslope direction. Fig 1 shows the location of the survey area and the survey line tracks relative to the New Jersey coastline. The towed streamer was 3000 m in length, with 240 receiver groups (channels) spaced 12.5 m apart, and a nominal source-to-near-group distance of 231.5 m. The source array was relatively small (for the *Langseth*), having four guns with a total volume of 700 in^3^. A sympathetic shuttle bounce on a spare source element appears to have occurred on many of the shots during the survey. The intershot spacing was 12.5 m, and both the source and the streamer were towed at a depth of 4.5 m. Line spacing was approximately 150 m. Data from lines 1 and 2 were removed because of faulty navigation and data from lines 21–23 were removed because of a potential problem with the source array. In this study we analyze 270,816 seismic shots from 74 lines, or nearly 65 million individual traces collected from 240 channels at a sample rate of 2 kHz, with each trace being 4.096 s (8192 samples) in length. Although a Nyquist filter removes signal energy above 824 Hz for these traces, previous work has shown that the energy contained in the signals above this frequency does not contribute significantly to the calculated received levels for mitigation purposes [9].

## Methods

### RMS and SEL levels

For every seismic shot analyzed, we calculate the received root-mean-square (RMS) sound pressure level (SPL) and sound exposure level (SEL) for each channel in the streamer. To calculate these values we follow the same procedure described in Crone and Tolstoy [11], which requires that we first high-pass filter each trace, adjust the received signal to account for the “group-length” effect, and then window the signal around the source pulse peak.

We high-pass filter each receiver trace using a sixth-order Butterworth filter with a passband frequency of 11 Hz (-1 dB) and a cuttoff frequency of 9.78 Hz (-3 dB) to remove low-frequency noise associated with streamer motion and ocean waves. This filter was shown to maximize the resulting estimates of RMS and SEL by removing long period signals associated with streamer noise, and minimizing the removal of signal energy associated with the source impulse [11].

Data from each channel on the streamer is generated not by a single hydrophone but by a group of hydrophones distributed along a length of the streamer in order to minimize the recording of horizontally traveling acoustic energy and maximize the recording of acoustic energy returning from the seafloor. To account for this “group-length” effect, we multiply received pressures by a factor of two. This group length adjustment factor was determined empirically using data from the Gulf of Mexico calibration study [9].

Finally we window each trace using a 4-s window centered on the source impulse peak. We use an initial window of this length to be certain that we are including the entire source signal pulse as well as short sections of noise on either side of the pulse. For some source configurations, in shallow water especially, reflected and refracted arrivals can increase the length of the received source pulse to well beyond one second, so a starting window of several seconds is required. Overly long initial windows are acceptable but short windows that truncate the source pulse must be avoided to minimize windowing bias [11].

We use the following equation to calculate the RMS SPL values in dB referenced to 1*μ*Pa for each prepared trace:

$$\begin{array}{c} RMS=10\log_{10}(\frac{1}{T_{90}f_{s}}\sum_{i=1}^{N}p_{i}^{2}) \end{array}$$ (1)

where *p*_*i*_ is the windowed signal of length N samples in units of micropascals, *f*_*s*_ is the sample rate (2000 Hz), and *T*_90_ is the length of the input signal (in seconds) that contains 90% of the cumulative power of the signal [7–9, 11, 24].

Sound exposure is defined as the square of the sound pressure signal integrated in time over the duration of the exposure [25], and SEL is this quantity expressed in decibels referenced to 1 *μ*Pa^2^ s. We use the following equation to estimate SEL for each prepared trace [26]:

$$\begin{array}{c} SEL=10\log_{10}(\frac{1}{T_{90}f_{s}}\sum_{i=1}^{N}p_{i}^{2})+10\log_{10}T_{90} \end{array}$$ (2)

Thus SEL, which is equivalent to the energy flux density, contains a term which accounts for the time over which a marine animal would be exposed to the sound.

### Mitigation radii

NMFS currently defines safety criteria (levels above which there is concern of auditory impairment or injury) for pinnipeds and cetaceans as 190 and 180 dB RMS referenced to 1 *μ*Pa, respectively. Additionally, the 160 dB level is identified as the level above which, in the view of NMFS, there is likely to be behavioral disturbance for cetaceans. Thus the distances beyond which the received levels fall below 180 and 160 dB are commonly referred to as the “mitigation radii”.

To estimate the distances to these mitigation radii for each seismic shot, we must first remove outlier RMS and SEL values which usually result from malfunctioning receiver groups on the streamer. We use the method described in Crone and Tolstoy [11] to identify the channels that are not functioning properly along each survey line separately to account for the possibility that the subset of improperly functioning receiver groups will change during the survey. The procedure involves averaging all data from each channel along each entire line, and removing those channels with average values that are significantly different (±2 dB) from neighboring channels, and those channels returning values with an unacceptably high shot-to-shot variance.

**Fig 2:**
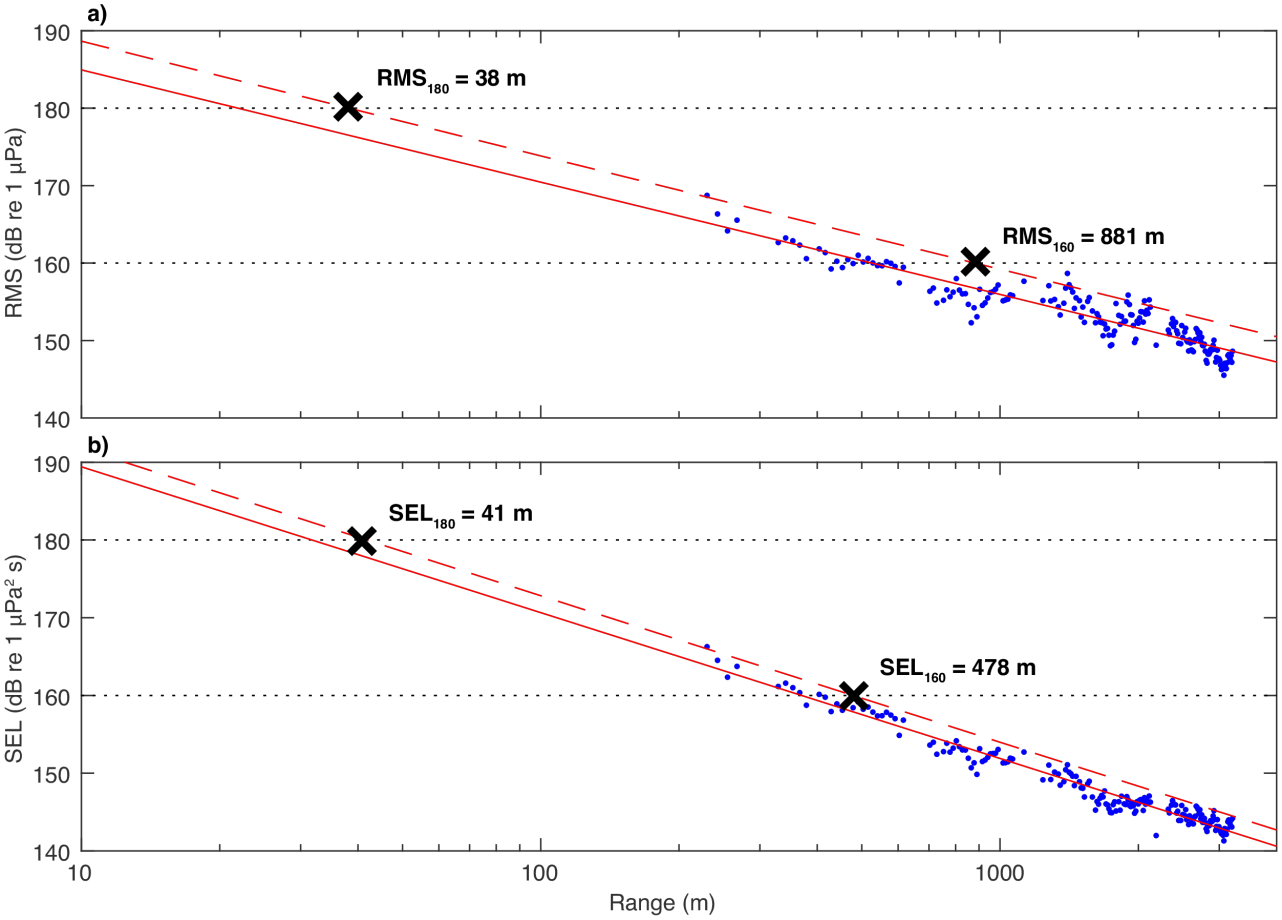
Typical data from a single shot showing the estimated (a) RMS and (b) SEL values measured along the streamer (blue dots) and the regression curves used to establish the 180 and 160 dB radii (red lines). The solid lines are the regression curves and the dashed lines are the 95% upper prediction bounds for these curves. The “X” markers indicate the intersections of the upper prediction bounds with the 180 and 160 dB levels and are labeled with the radii values.

We are then able to estimate the distances to the mitigation radii by fitting a curve to the remaining RMS and SEL levels, and determining the 95% upper prediction bounds for these curves. We use a two-term regression model in the following form:

$$\begin{array}{c} y=a_{1}+a_{2}\log_{10}(x) \end{array}$$ (3)

where *x* is range, which results in very good fits to the data. Fig 2 shows representative examples of these curves and the 95% prediction bounds for the RMS and SEL values from a single shot. The 180 and 160 dB radii obtained using the RMS and SEL values, denoted RMS_180_, RMS_160_, SEL_180_, and SEL_160_, are determined by finding the intersection of the respective 95% prediction bound curve with the 180 and 160 dB levels.

**Fig 3:**
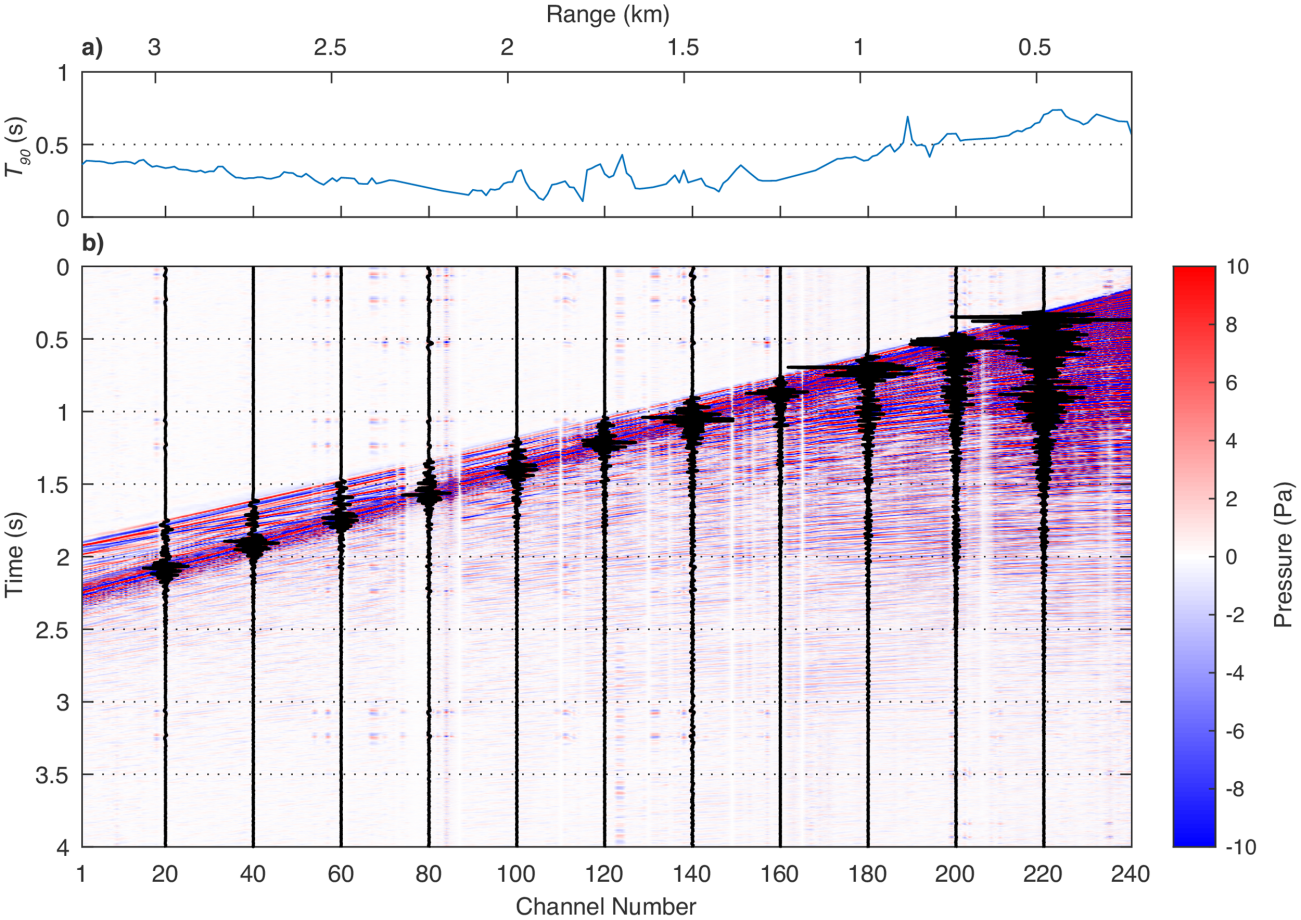
(a) Plot of *T*_90_ for a typical seismic shot and (b) colored contours of pressure for the same shot gather overlain with a subset of waveform traces. Distance to the near group for this shot (channel 240) is 230.1 m. The length of the source pulse is greatest at the near group, and diminishes with range for about 2–2.5 km. A slight increase in the received pulse length occurs because a refracted arrival is recorded coincident with the direct arrival.

Fig 3 shows a plot of *T*_90_ as a function of range and contours of pressure (shot gather) for the same seismic shot. In general, the longest pulse lengths are typically recorded at the near group, and the values of *T*_90_ diminish with range, although refracted arrivals can increase the pulse length near the very end of the streamer. Unlike shots from the 36-gun 6600 in^3^ four string source array [11], the source used in this survey only rarely generates a pulse that is longer than 1 s (0.7% of traces). The diminishing value of *T*_90_ with range is why the SEL values in Fig 2 fall off more rapidly than the RMS values, and this is why the SEL values can produce a value for SEL_160_ which is significantly smaller than RMS_160_ while at the same time producing values of RMS_180_ and SEL_180_ which are quite similar.

### Seafloor bathymetry and slope

During the cruise continuous multibeam data were collected using a 12 kHz Kongsberg EM122 1×1 degree sonar system in dual-ping shallow-water mode. Sound velocity profiles were obtained approximately daily with expendable bathythermograph (XBT) measurements using Sippican T-4 XBT probes, which are sensitive to temperature fluctuations on the order of 0.1°C. The multibeam swath width was constrained to ~400 m, resulting in swath overlaps ranging from ~10–63%, and an along-track beam spacing that was approximately 1 m [27]. This constrained swath width minimized errant returns from the outside beams and reduced the need for manual ping editing.

We cleaned, tide-corrected, processed, and gridded the multibeam data using MB-System v5.5.2267 [28] and Generic Mapping Tools (GMT) v5.2.1 [29]. Shipboard processing included the conversion of raw Kongsberg files to MB-System files and the generation of ancillary files. The ancillary files facilitated data QC and provided fast access to metadata as well as navigation and two-way travel times for each beam. Post-cruise processing involved automatic beam flagging (i.e. removal) using criteria set for the survey. Along track beams were flagged if they fell ±10% outside of the median depth, while across track beams were flagged if the beam-to-beam spacing exceeded ±10% of the median depth. Additionally, beams were flagged if the beam-to-beam angle exceeded 15 degrees. All bathymetry data were tide corrected using the OTPS2 tide correction software, which supplements a low-resolution global model with a high-resolution local model via the TPXO.8 atlas [30]. The edits and tide correction were then applied in the ray tracing algorithm during the processing step. Finally, we gridded the bathymetry data at 5 m^2^, and then regridded on the 600×60 grid shown in this study. We calculate the seafloor slope in the along-track direction (i.e. dz/dy) using a third-order finite difference estimator which uses six points to estimate the slope at each point [31, 32].

**Fig 4:**
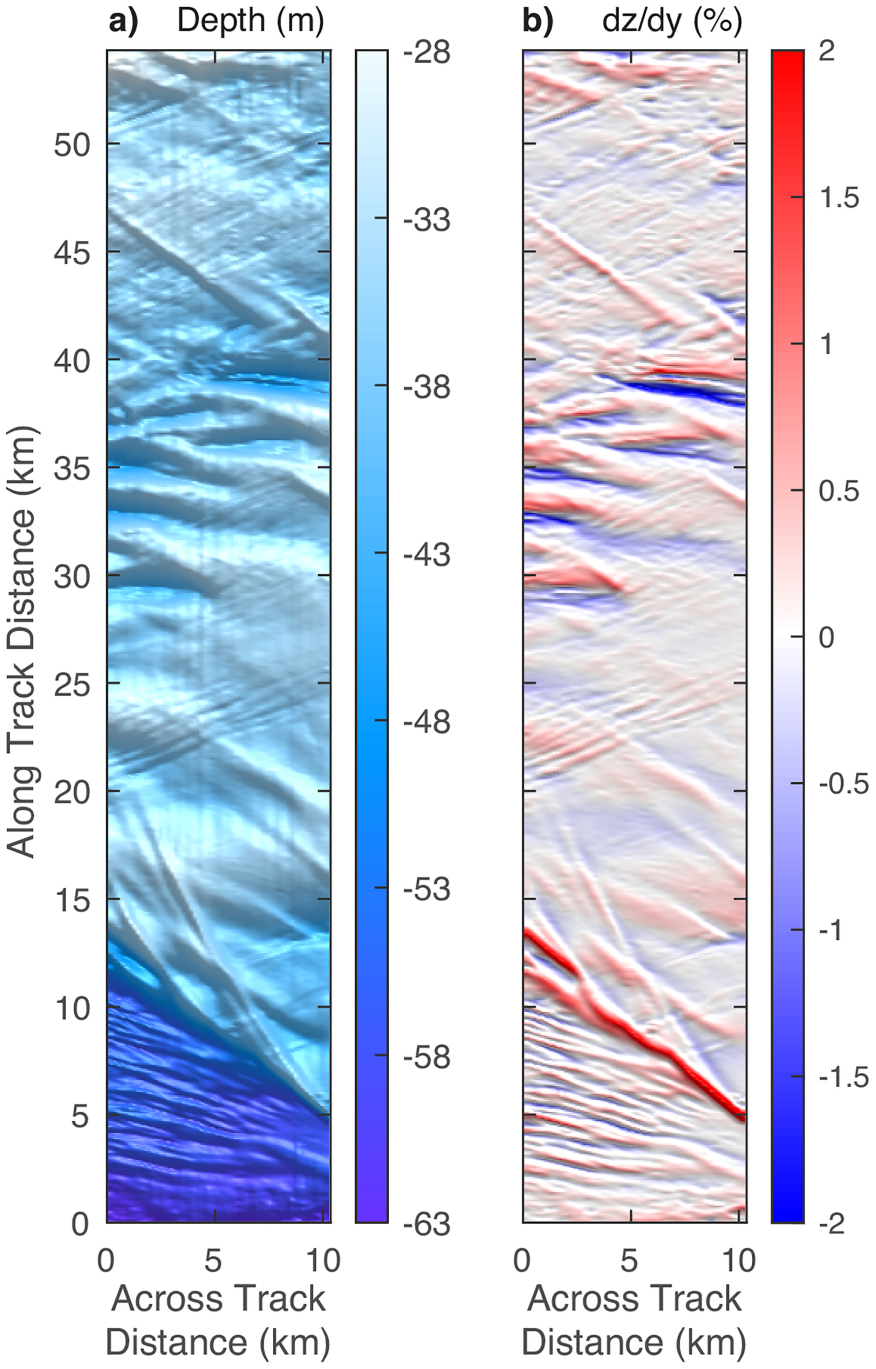
Maps of (a) depth and (b) slope in the along-track direction, within the survey area.

Fig 4 shows maps of the bathymetry and the slope in the along-track direction of the seafloor within the survey area. The mid-shelf scarp is a dominant feature in both maps, where depth changes by about 20 m and the slopes are off scale (as high as 5%) along parts of the feature. Several of the sand dunes (between 30 and 40 km along track) also have relatively large slope values.

## Results and discussion

**Fig 5:**
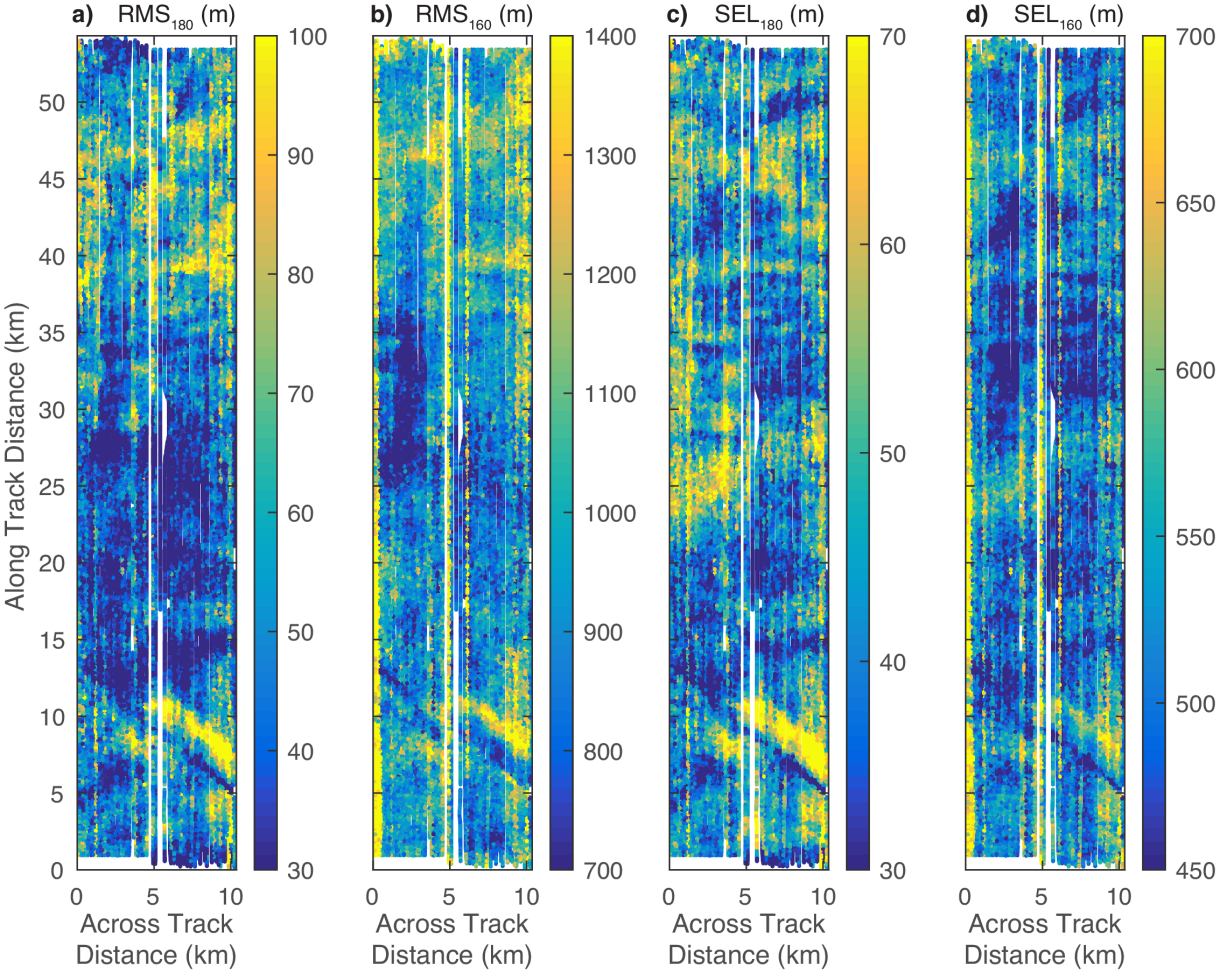
Maps of (a) RMS_180_, (b) RMS_160_, (c) SEL_180_, and (d) SEL_160_ within the survey area. For clarity each of these maps is shown with a separate color scale. These maps are generated using color-coded scatter plots of the actual estimated radii for each seismic shot rather than by interpolating and contouring. Overplotting biases are reduced by setting the layer order of each dot randomly.

Fig 5 shows maps of the four estimated mitigation radii values within the survey area in the same map view shown in Fig 4. Mitigation radii estimates are affected in part by seafloor depth, but appear to be more strongly affected by seafloor slope, especially near the mid-shelf scarp and near the dunes with the greatest slope values. Other variability could be related to bottom type and sub-bottom characteristics, but detailed information on bottom type that would be required for such interpretations are not available at this time. Mitigation radii values are different for lines that were surveyed in the upslope versus downslope directions, an effect that we explore in greater detail below using three-dimensional interpolated volumes of the raw RMS and SEL values.

**Fig 6:**
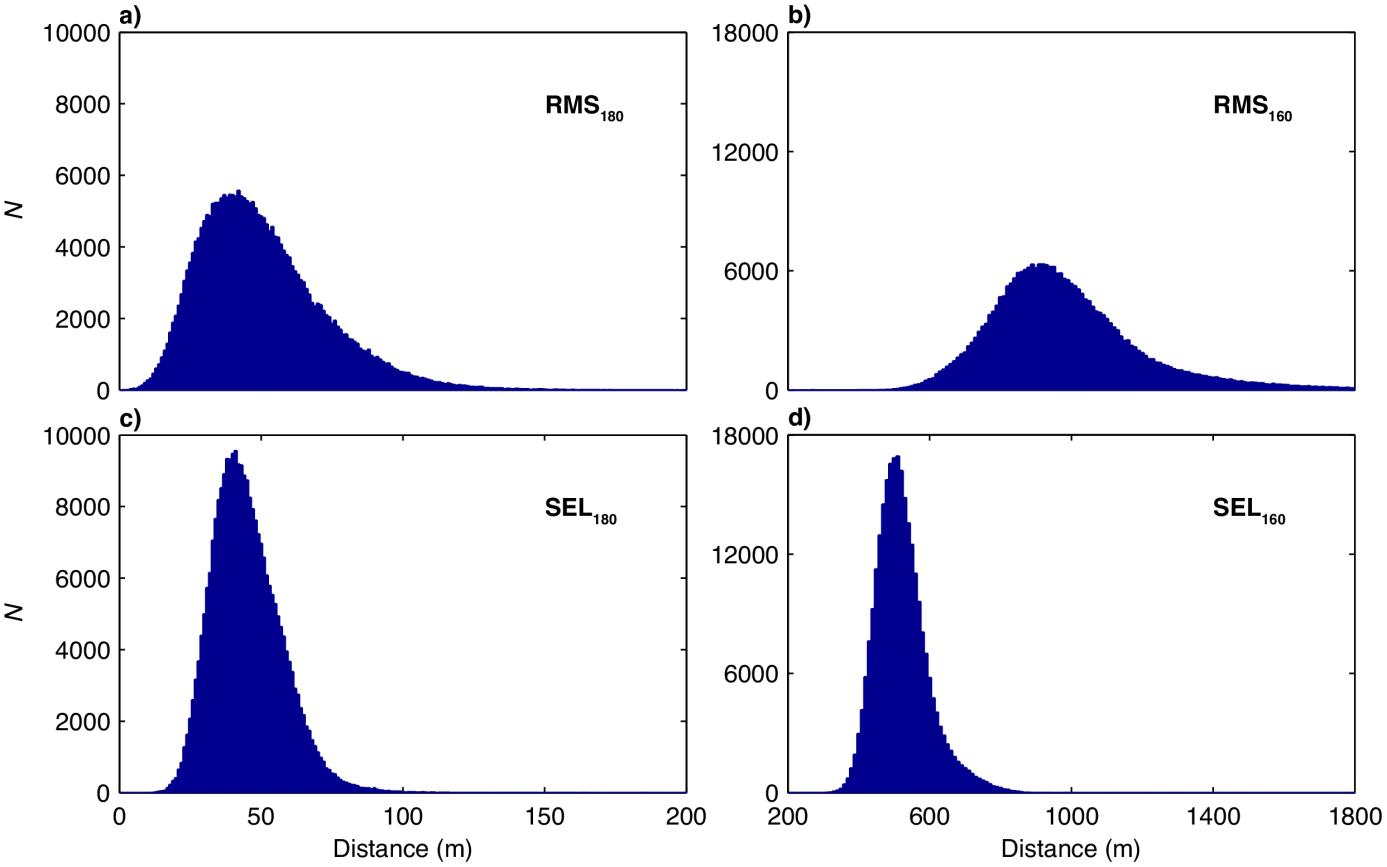
Histograms of (a) RMS_180_, (b)RMS_160_, (c) SEL_180_, and (d) SEL_160_ values for all 270,816 seismic shots analyzed in this study. Median and 95th percentile values are shown in Table 1.

Fig 6 shows histograms of the four estimated mitigation radii for all 270,816 seismic shots analyzed in this study. Table 1 shows a set of statistics for these distributions along with the predicted 180 and 160 dB mitigation radii that were used during the survey. The median values for RMS_180_ and SEL_180_ are similar at 47 and 44 m, respectively. This result is expected considering the reduction of *T*_90_ discussed in reference to Figs 2 and 3. The median value for RMS_160_ is 951 m, and for SEL_160_ is 513 m. This difference is also expected considering the small average value of *T*_90_ overall. The predicted mitigation radii that were used during the survey in compliance with NMFS permitting are approximately 4–8 times greater than the 95th percentile values for the measured radii. These results are reasonable considering the highly conservative approach used for *Langseth* marine mammal mitigation efforts, and are also consistent with the results of previous efforts to estimate mitigation radii using the towed seismic streamer [10, 11].

**Table 1:** Median and 95th percentile distances (in m) to the 180 and 160 dB mitigation levels determined using RMS and SEL. The predicted mitigation radii used during the survey are shown in the last column.

	RMS			SEL			Predicted
	*Median*	*STD*	*95%*	*Median*	*STD*	*95%*	
**180 dB**	47	22	92	44	12	67	378
**160 dB**	951	225	1408	513	75	663	5240

**Fig 7:**
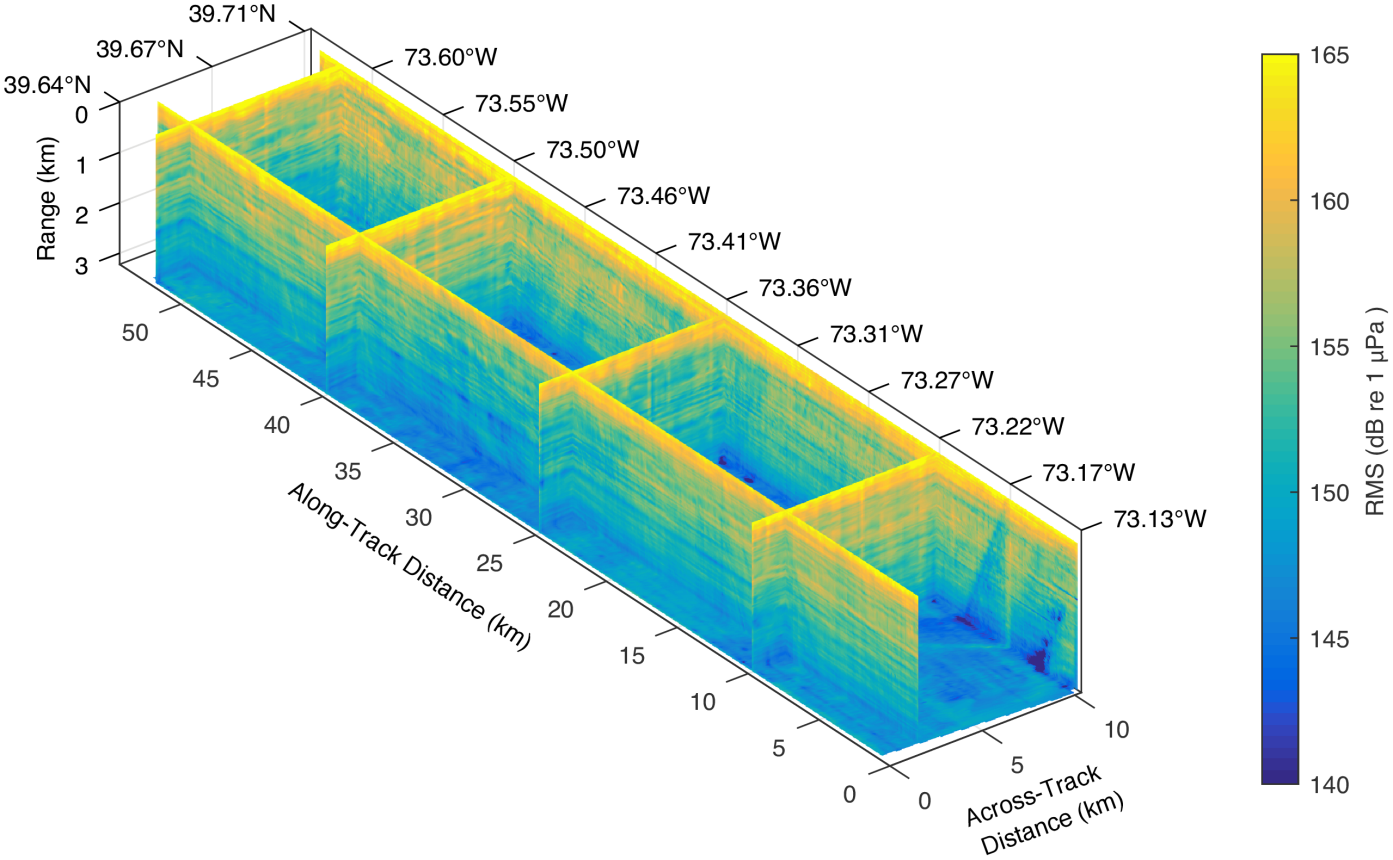
Selected slices through the three-dimensional interpolated volume of the estimated RMS levels within the survey area as a function of distance along and across the survey tracks, and as a function of distance from the seismic source, here depicted in the vertical direction. Latitude and longitude tick labels apply to the side of the volume labeled. A similar volume was generated for the SEL values. Strong apparent correlations between slices in the across-track direction suggest that the much of the variance is controlled by the seafloor and subseafloor properties, including the seafloor depth, slope, bottom type, and subseafloor sedimentary structure.

**Fig 8:**
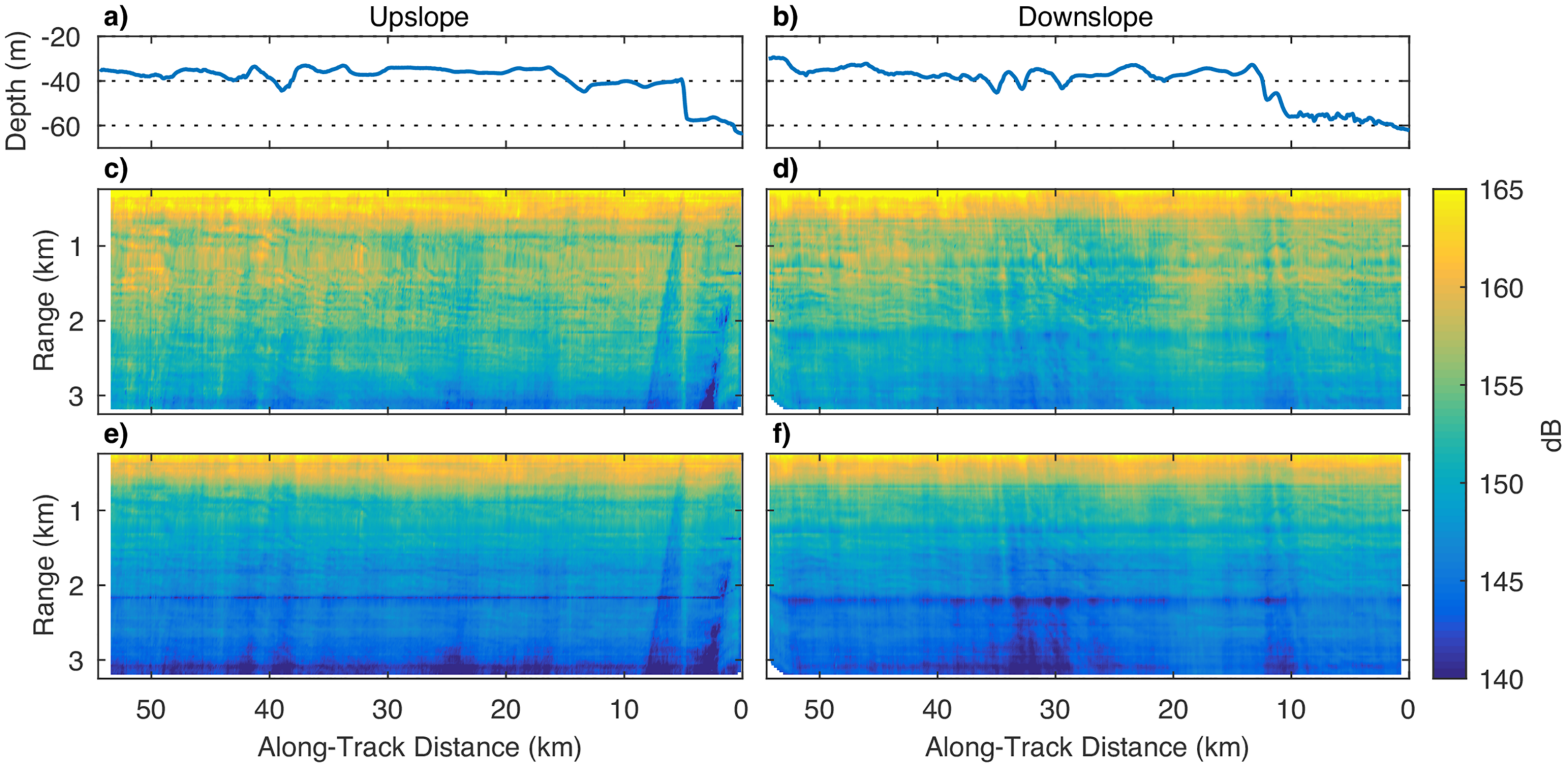
(a and b) Seafloor depth, and representative along-track slices through the the three-dimensional interpolated volume of (c and d) the RMS levels (in dB re 1 *μ*Pa), and (e and f) the SEL levels (in dB re 1 *μ*Pa^2^ s). The panels on the left-hand side show interpolated data collected while the ship was traveling in the upslope direction. The panels on the right-hand side show data collected in the downslope direction.

To explore the relationship between seafloor slope, survey direction, and the measured RMS and SEL levels, we constructed a three-dimensional interpolated volume of these values. Fig 7 shows an oblique view of several slices through the RMS volume, and Fig 8 shows four along-track slices through the two volumes, one in the upslope direction and one in the downslope direction. Variability in the values of RMS and SEL associated with changes in seafloor slope manifests itself in what could be called the “fabric” of the contour plots which change direction with changing survey direction. The effect is most pronounced at the mid-shelf scarp in the upslope direction. When the ship tows the seismic source over this scarp in this direction, the entire streamer falls into a “shadow zone” that results from interactions of the reverberating seismic energy with the scarp slope. As the streamer is towed into shallower water, streamer channels emerge from the shadow zone starting with those closest to the source. While the streamer is partway over the scarp, the lower RMS and SEL values on the more distal channels bias the estimated radii toward larger values, especially for the 180 dB radii, because of the change in slope of the regression curves. The opposite effect occurs when the ship is transiting in the downslope direction, but the effect does not appear to be as pronounced.

This process of shadowing RMS and SEL values and its interaction with the regression curves explains much of the variation in the estimated mitigation radii at the mid-shelf scarp shown in Fig 5. Critically, some of the variation, especially the increases in the estimated radii as the streamer passes over the scarp in the upslope direction are artifacts of the regression model. As distal channels fall into the shadow zone, the regression curve tilts increasingly downward, artificially increasing estimates of the 180 dB radii. Some of the variation is of course real, especially the reductions in the radii as the source first approaches the slope. However teasing apart the real and artifact effects will require a study involving an array of moored hydrophones in a shallow-water environment with scarps. In this study, the number of seismic shots resulting in large overestimates of the mitigation radii are relatively small, and should be offset by mitigation radii underestimates. The average and 95th percentile values for these radii shown in Table 1 remain robust.

## Conclusions

1. Analysis of field data from the *R/V Marcus G. Langseth’s* towed seismic streamer continues to provide important insights into the acoustic radiation patterns of the seismic source in shallow-water environments.
2. During the 2015 New Jersey central shelf survey, estimated distances to the 180 and 160 dB mitigation levels using streamer data were far shorter than the distances predicted prior to the cruise using extrapolations of calibration data. Predicted distances were 4–8 times greater than actual distances.
3. Some of the variability in received levels results from interactions between the acoustic energy and the variable seafloor slope, and artifacts in the regression model used to estimate mitigation radii outside the streamer range can result. For this reason care must be taken when interpreting outliers.
4. An acoustic radiation study using a moored hydrophone array would help to better resolve the three-dimensional acoustic field generated by a seismic source in shallow water, and would allow a fuller characterization of the strengths and limitations of estimating mitigation radii using a towed streamer. Such a study would aid in the development of an automated streamer-based real-time mitigation system.
